# Spontaneous control of HIV-1 viremia in a subject with protective HLA-B plus HLA-C alleles and HLA-C associated single nucleotide polymorphisms

**DOI:** 10.1186/s12967-014-0335-6

**Published:** 2014-12-05

**Authors:** Marco Moroni, Silvia Ghezzi, Paolo Baroli, Silvia Heltai, Davide De Battista, Simone Pensieroso, Mariangela Cavarelli, Stefania Dispinseri, Irene Vanni, Claudia Pastori, Pietro Zerbi, Antonella Tosoni, Elisa Vicenzi, Manuela Nebuloni, Kim Wong, Hong Zhao, Sarah McHugh, Guido Poli, Lucia Lopalco, Gabriella Scarlatti, Roberto Biassoni, James I Mullins, Mauro S Malnati, Massimo Alfano

**Affiliations:** Infectious Disease Unit, Busto Arsizio Public Hospital, P.le Solaro n. 3, Busto Arsizio, 21052 Varese Italy; Viral Pathogens and Biosafety Unit, Division of Immunology, Transplantation and Infectious Disease, San Raffaele Scientific Institute, Milan, Italy; Service Lab Fleming Research, Busto Arsizio, Varese Italy; Human Virology Unit, Division of Immunology, Transplantation and Infectious Disease, San Raffaele Scientific Institute, Milan, Italy; Viral Evolution and Transmission Unit, Division of Immunology, Transplantation and Infectious Disease, San Raffaele Scientific Institute, Milan, Italy; Department of Translational Research, Istituto Giannina Gaslini, Genoa, Italy; Immunobiology of HIV Unit, Division of Immunology, Transplantation and Infectious Disease, San Raffaele Scientific Institute, Milan, Italy; Pathology Unit, Luigi Sacco Hospital, Department of Biomedical and Clinical Sciences, University of Milan, Milan, Italy; Departments of Microbiology, Medicine and Laboratory Medicine, University of Washington, Seattle, WA USA; AIDS Immunopathogenesis Unit, Division of Immunology, Transplantation and Infectious Disease, San Raffaele Scientific Institute, Via Olgettina n. 58, Milan, 20132 Italy; School of Medicine, Vita-Salute San Raffaele University, Milan, Italy; Present address; Division of Experimental Oncology, Unit of Urology, URI; IRCCS Ospedale San Raffaele, Via Olgettina n. 60, Milan, 20132 Italy

**Keywords:** Human Immunodeficiency Virus (HIV), Viremia, Human Leukocyte Antigen (HLA), Single Nucleotide Polymorphisms (SNPs), Elite controller (ELC), Long term nonprogressor (LTNP)

## Abstract

**Introduction:**

Understanding the mechanisms by which some individuals are able to naturally control HIV-1 infection is an important goal of AIDS research. We here describe the case of an HIV-1^+^ woman, CASE1, who has spontaneously controlled her viremia for the last 14 of her 20 years of infection.

**Methods:**

CASE1 has been clinically monitored since 1993. Detailed immunological, virological and histological analyses were performed on samples obtained between 2009 and 2011.

**Results:**

As for other Elite Controllers, CASE1 is characterized by low to undetectable levels of plasma HIV-1 RNA, peripheral blood mononuclear cell (PBMC) associated HIV-1 DNA and reduced *in vitro* susceptibility of target cells to HIV-1 infection. Furthermore, a slow rate of virus evolution was demonstrated in spite the lack of assumption of any antiretroviral agent. CASE1 failed to transmit HIV-1 to either her sexual male partner or to her child born by vaginal delivery. Normal values and ratios of T and B cells were observed, along with normal histology of the intestinal mucosa. Attempts to isolate HIV-1 from her PBMC and gut-derived cells were unsuccessful, despite expression of normal cell surface levels of CD4, CCRC5 and CXCR4. CASE1 did not produce detectable anti-HIV neutralizing antibodies in her serum or genital mucosal fluid although she displayed potent T cell responses against HIV-1 Gag and Nef. CASE1 also possessed multiple genetic polymorphisms, including HLA alleles (B*14, B*57, C*06 and C*08.02) and HLA-C single nucleotide polymorphisms (SNPs, rs9264942 C/C and rs67384697 del/del), that have been previously individually associated with spontaneous control of plasma viremia, maintenance of high CD4^+^ T cell counts and delayed disease progression.

**Conclusions:**

CASE1 has controlled her HIV-1 viremia below the limit of detection in the absence of antiretroviral therapy for more than 14 years and has not shown any sign of immunologic deterioration or disease progression. Co-expression of multiple protective HLA alleles, HLA-C SNPs and strong T cell responses against HIV-1 proteins are the most likely explanation of this very benign case of spontaneous control of HIV-1 disease progression.

**Electronic supplementary material:**

The online version of this article (doi:10.1186/s12967-014-0335-6) contains supplementary material, which is available to authorized users.

## Introduction

In the absence of combination antiretroviral therapy (cART) HIV-1 infection results in AIDS and death in most individuals. In contrast, a minority of individuals demonstrate an almost absolute capacity to resist infection (e.g., carriers of CCR5-∆32 homozygosity) [1]. Others, when infected, experience significantly delayed disease progression, either in terms of maintenance of peripheral CD4^+^ T cell counts ≥500 cells/μl after 7–8 years of HIV-1 infection (long-term non progressors, LTNP) or by spontaneously controlling their HIV viremia [commonly referred to as “Elite Controllers (ELC)” when 90% of plasma HIV-1 RNA values are <50 copies/ml, or “HIV Controllers (HIC)”, when 90% of plasma viremia measurements are <500 copies/ml for ≥12 months]. Rare (<1% of all infected individuals) cases show both LTNP and EC features and have been defined as “elite LTNP” [2,3].

Among other correlates of delayed HIV-1 progression in the absence of cART the role of several alleles of Human Leukocyte Antigen (HLA) Class I genes, such as HLA-B*27 [4] and HLA-B*57 [5] has been well established. Additional associations between MHC-Class I and III SNPs and the LTNP phenotype have been observed [6].

Thus, CASE1 detailed immunologic, virological and genetic profile may provide clues to the design of therapeutic vaccines aiming at achieving a functional cure of HIV-1 infection in the absence of ART [7].

## Methods

### IRB approval

Biological samples were collected after receiving formal written waiver from the institutional review board (protocol MUCIM approved in January 2007 by the Ethics Committee Ospedale San Raffaele, Milan, Italy), and signed written informed consent.

### HIV-1 RNA and DNA quantitation

HIV-1 plasma RNA was measured using the Amplicor Monitor (Roche) assay (dynamic range: 50–750,000 copies/mL), and HIV-1 DNA quantification was performed using an in-house real-time PCR assay, as described [8].

### Viral genetic analysis

Isolation of plasma HIV RNA and PBMC-associated HIV DNA, reverse transcription, amplification, *gag* and *env* sequencing were performed according to the published methods reported in the Additional file 1 section.

### MiR-148a/b binding site (single nucleotide polymorphisms, SNP: rs67384697) and -35Kb 5′UTR HLA-C (SNP: rs9264942) analysis

Genomic DNA was extracted from CASE1’s PBMC using the PureLink Genomic DNA kit (Invitrogen, Carlsbad, CA), and a pyrosequencing approach was used to determine SNPs. Detailed methods are reported in the Additional file 1 section.

### Culture and co-culture of rectal biopsy with allogeneic T cell blasts

Histocultures of intestinal biopsies were performed as previously reported [9]; both cells and histoculture supernatants were collected 24 h later. Cells were dispersed by enzymatic digestion with collagenase IV (0.5 mg/ml in complete culture medium, 30 min at 37°C), passed through a 22G needle and filtered with a 70 μm cell strainer. The digestion was repeated and cells from the two rounds were pooled and debris removed by centrifugation. Two million biopsy-derived cells were cultivated either alone or with 2x10 [6] PHA-stimulated PBMC from two different donors that had been previously depleted or not of CD8^+^ T cells by magnetic immunobeads. Both cultures and co-cultures were maintained for 30 days in IL-2 enriched medium, collecting their supernatants every 3 days for measurement of virus using either Mg^++^-dependent reverse transcriptase (RT) activity [10] or HIV-1 p24 Gag antigen by ELISA.

### HIV-1 isolation from and *ex-vivo* infection of CASE1 PBMC

Three independent attempts were made to isolate HIV-1 from peripheral CD4^+^ T cells according to published protocols [11]. Supernatants were collected every 3–4 days for up to 4 weeks of cultivation and tested for the presence of either Mg^++^-dependent reverse transcriptase (RT) activity or HIV-1 p24 Gag antigen by ELISA.

For *ex-vivo* infection, CD4^+^ leukocytes from both CASE1 and her partner were isolated by negative selection from peripheral blood by Ficoll-Hypaque, washed and suspended in complete medium and purified as described above. Cells were stimulated with PHA and 3 days later washed and infected with CCR5-dependent (R5) HIV-1_BaL_ or CXCR4-dependent (X4) HIV-1_LAI/IIIB_ at a multiplicity of infection of 0.2. Culture supernatants were collected every 3–4 days for up to 4 weeks and tested for the presence of RT activity.

### ELISpot assay for IFN-γ

#### Peptides

The Variable Overlapping Peptide Scanning Design (VOPSD) technique [12] was used to design peptides derived from HIV-1 encoded antigens Tat (11 peptides, Repository number: ARP7103.1-11), Nef (30 peptides, Repository number: ARP7102.1-30) and Gag (84 peptides, Repository number: ARP7114.1-84) kindly provided by the Centre for AIDS Reagents, National Institute for Biological Standards and Control (NIBSC HPA UK). Single peptides or peptide pools were used to stimulate PBMC collected in November 2009 and June 2011 at a final concentration of 2 μM. Validation of the VOPSD strategy was obtained by direct comparison with 15mer or 20mer peptide sets, as recently reported [12]. ELISpot for IFN-γ was performed as previously described [13], and detailed methods reported in the Additional file 1 section.

### Intracellular cytokine staining

Thawed PBMC (≥80% viable) were plated in a 96-well plate after 4 h of resting (EuroClone) at a concentration of 1x10 [6] PBMC/well in complete RPMI medium [10% FBS (Lonza-BioWhittaker) in RPMI (Lonza-BioWhittaker)] with single/pools of HIV-1 derived peptides (2 μM) in the presence of a mixture of co-stimulatory anti-CD28 and anti-CD49d Ab (1.3 μg/ml each, Becton Dickinson). Cells were then treated and stained as previously described [13]. Detailed methods are reported in the Additional file 1 section.

## Results

### Clinical history

**Figure 1:**
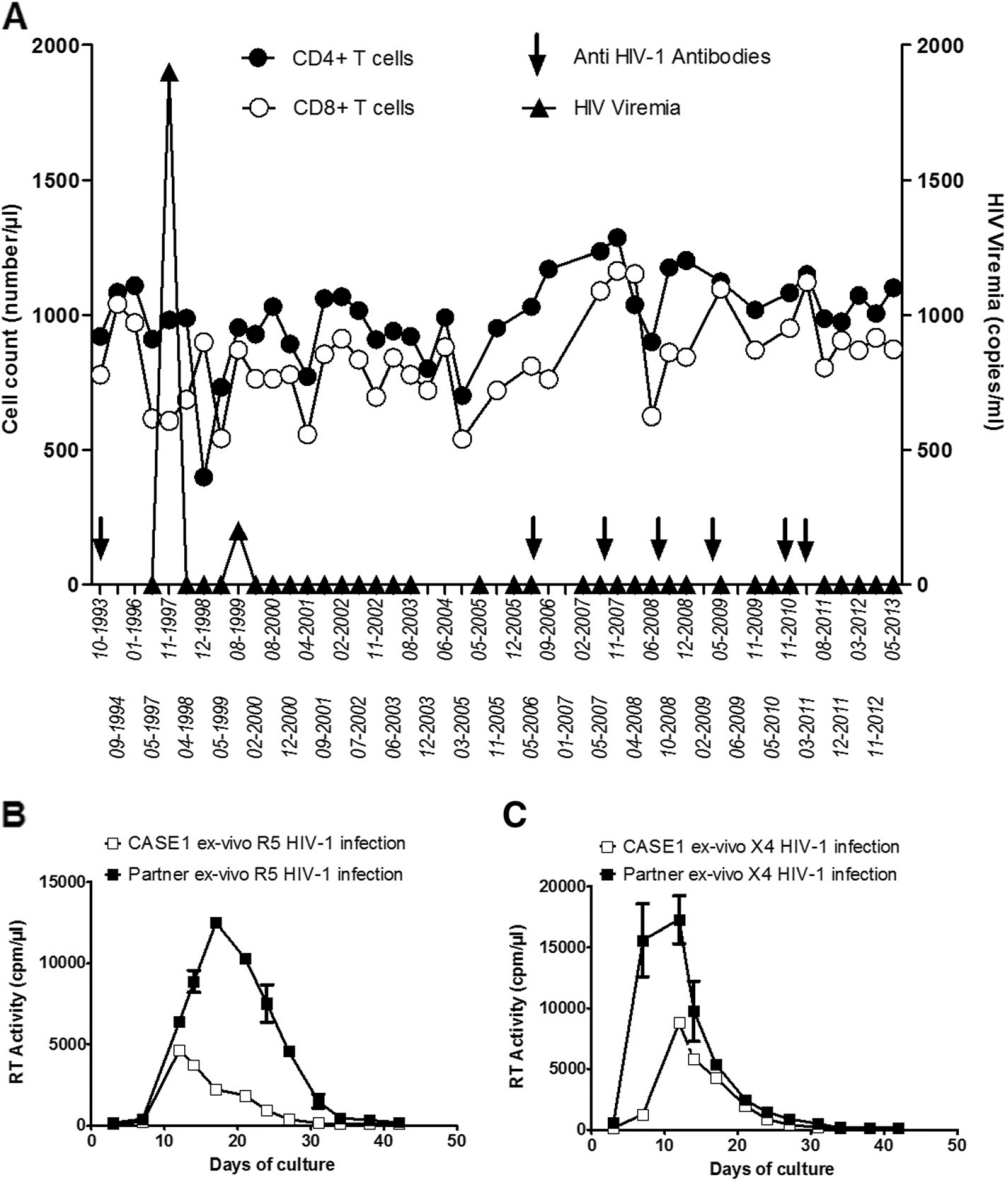
**CASE1’s natural control of HIV-1 viremia. (A)** Levels of peripheral CD4^+^ and CD8^+^ T cells, plasma viral RNA and the presence of anti-HIV-1 Ab in CASE1 plasma were monitored over 20 years of infection. Purified peripheral CD4^+^ cells were isolated from CASE1 and infected with either an R5 **(B)** or an X4 **(C)** strain, resulting in detectable virus replication. Purified CD4^+^ T cells of her sexual partner supported higher levels of virus replication. Bars indicate error of the mean from duplicate cultures.

A previous female intravenous-drug user, (CASE1), was diagnosed with HIV-1 infection in 1993 (age: 23 years old) in the course of a pregnancy screening test; she was confirmed to be HIV-1^+^ in the following years. CASE1 was repeatedly found seropositive for anti-HIV-1 antibodies (Ab), but negative for anti-HIV-2 Ab. Her viremia levels were undetectable when assessed for the first time in May 1997, but 1,900 copies of HIV-1 RNA/ml were demonstrated in Nov 1997 that dropped to 200 copies/ml in Aug 1999. Since then, HIV-1 viremia levels, that were monitored at least twice a year, have remained <50 copies/ml although CASE1 never assumed any antiretroviral agent. She also never reported clinical episodes suggestive of immunodeficiency, while her peripheral CD4^+^ and CD8^+^ T cell counts have remained in the normal range of uninfected healthy individuals through all these years (Figure 1A).

CASE1 reported >20 years of unprotected sexual intercourse with her male partner, who remained HIV-1 seronegative. She also had a child born in 1994 by vaginal delivery who has remained uninfected.

In 1997 CASE1 was diagnosed with hepatitis C virus (HCV, genotype 3a) infection, becoming negative for HCV viremia with normal levels of transaminases in 2005 after therapy with Pegylated Interferon plus Rebetol (Additional file 1: Figure S1).

### Virological determinations and *ex vivo* infection of CASE1 CD4^+^ T cells

In June 2009 colon biopsies were taken for diagnostic purposes. Hematoxylin-eosin, Giemsa, PAS staining and transmission electron microscopy revealed normal histology of the intestinal mucosa, lack of inflammatory infiltrates, microbes, virions or cell injury (Additional file 1: Figure S2). The tissue was also negative for the presence of HIV-1 p24 Gag^+^ cells by immunohistochemistry, although its viral DNA content was estimated to be 881 copies/10^6^ cells. *In vitro* cultivation of either 6 colon biopsy histocultures or of colon-derived leukocytes (2x10^6^ cells) co-cultivated with CD8-depleted T cell blasts of two seronegative donors were negative for virus replication in terms of either RT activity or p24 Gag production (data not shown). Furthermore, neither allogeneic cultivation of 5x10^5^ PBMC-purified CD4^+^ cells from CASE1 with T cell blasts of two seronegative donors, nor direct cultivation of the same number of CASE1 cells in medium containing IL-2, induced virus replication (data not shown).

*Ex vivo* infection of CASE1 CD4^+^ T cell blasts with HIV-1 R5_BaL_ or X4_LAI/IIIB_ strains resulted in virus replication, although at lower levels compared to those observed after infection of CD4^+^ T cell blasts from her partner (Figure 1B and C).

### Sequence analysis of CASE 1 HIV-1 DNA

Quantitative PCR from CASE1 plasma samples were repeatedly <50 copies HIV-1 RNA/ml (Figure 1A), and only 2 viral genomes were detected in a total of 9 mL of plasma from June and Nov 2009 by multiplex PCR to amplify both full length *gag* and *gp120 env* coding sequences (see Additional file 1). Her PBMC-associated HIV-1 DNA load in June, and Nov 2009 and in June 2011 was 28, <13 and 10 copies/10^6^ cells, respectively. In the same dates, 23 *gag-* and 20 *gp120 env*- sequences (all with open reading frames) were obtained from a total of 21x10^6^ PBMC.

**Figure 2:**
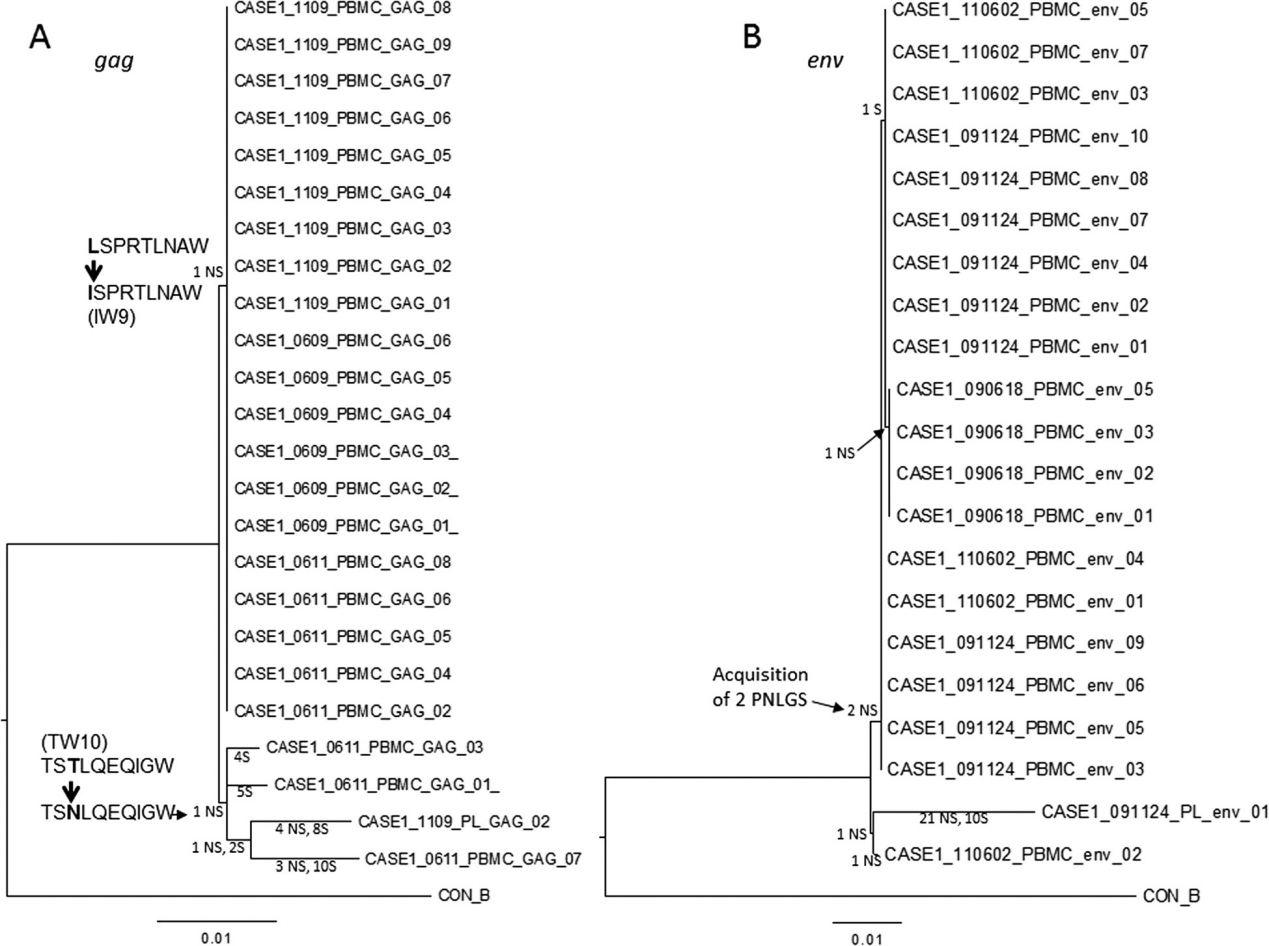
**Phylogenetic analysis of CASE1 HIV-1** ***gag*** **and** ***env*** **sequences.** Viral genome templates corresponding to the near full length *gag* gene (HXB2 nucleotides 818–2276) and the gp120 coding region of *env* (HXB2 nucleotides 6229–7786) were PCR amplified from blood plasma and PBMC DNA collected in June and November of 2009, and from PBMC from June 2011, as described in Additional data. PCR products derived from individual viral templates were sequenced directly and analyzed as described in Additional data. PBMC- and plasma (PL)-derived sequences are labeled by date (YYMMDD). Mutations at specific tree branches are tallied as either non-synonymous (NS) or synonymous (S). Two epitopes changed over time in *gag* **(A)**. The B*57 associated AA substitution I147L in the immunodominant B*57 epitope IW9 was a reversion mutation, resulting in a susceptible, consensus form of the epitope - hence the branch extends from the CASE1 sequences earlier in infection towards the consensus. The TW10 mutation, by contrast, was an escape mutation, with the consensus of all of the Group M viruses being the susceptible form of the epitope - hence the branch extends from the CASE1 sequences earlier in infection away from the consensus. These 2 NS mutations are bolded and noted by a thick arrow between the sequences. NS mutations resulting in two potential N-linked glycosylation sites (PNLGS) were found in *env* **(B)**. The scale bar below each dendrogram illustrates the branch length formed by mutations corresponding to a 1% change. Amino acid alignments are provided in Additional data.

All 15 PBMC-derived single genome HIV-1 *gag* coding sequences from June (N = 6) and Nov (N = 9) 2009 were identical, as were 5 out of 8 sequences from June 2011 (Figure 2A). In the case of *env* (Figure 2B), all 4 PBMC sequences from June 2009 were identical; all 10 sequences from Nov 2009 encoded identical proteins (4 sequences had the same synonymous site mutation) and differed from the June 2009 population at only one amino acid. Three out of 6 *env* sequences from June 2011 were identical to the major Nov 2009 population and 2 out of 6 were identical to the minor Nov 2009 population. One *env* sequence and *gag* sequences from 3 June 2011 were subtle outliers, with an additional 5 or 5–17 changes, respectively, relative to the major virus populations (Figure 2). Despite this exceptional overall homogeneity in cell-associated viral DNA (Mean sequence diversity across all 3 time points =0.17% in *gag* and 0.08% in *env*), the single *gag* and *env* sequences obtained from the Nov 2009 plasma sample were significantly divergent from the PBMC-associated DNA sequences (Mean =1.1% in *gag* and 2.5% in *env*), although they each shared some of the mutations that distinguished the PBMC outlier sequences from Nov2011. The changes in *gag* shared with each of the outlier PBMC led to the reversion of the B*57-associated AA substitution I147L in the immunodominant [14] B*57 epitope IW9, restoring the optimal CTL epitope sequence, and to the introduction of the T242N substitution in the TW10 epitope (Figure 2A), normally associated with CTL escape. No mutations were observed in the B*57-restricted epitopes KF11 or the B*14 epitope DA9 (Additional file 1: Figure S3A). Finally, 2 of the 3 shared non-synonymous site changes in *env* corresponded to acquisition of potential N-linked glycosylation sites (Figure 2B).

### CASE1 protective alleles

Although not carrying the protective alleles CCR5-∆32 and CCR2-64I, CASE1 does express HLA B and C alleles (B*14.02 and B*57.08; C*06 and C*08.02), previously associated, either alone [5,15] or in combination [16,17], with slow/non-progression in LTNP and low levels of viremia in the absence of cART assumption.

In addition, CASE1 possesses the C/C genotype in the rs9264942 (C/T) -35 SNP and the del/del polymorphism (rs67384697 G/deletion) mapping upstream to the 5′ and within the 3′ untranslated region of the HLA-C gene, respectively. These 2 SNPs have been reported to be in strong linkage dysequilibrium being associated with control of HIV-1 viremia [5,6].

### Immunological phenotypes and immune responses

CASE1 and her partner were found to have a distribution of B lymphocyte subpopulations comparable to those of uninfected individuals (Additional file 1: Table S1). Two serum samples (tested as total and purified immunoglobulins) and cervico-vaginal derived Ab collected in June 2009 and Nov 2009 did not show neutralizing activity against a panel of different HIV clades and phenotypes (Additional file 1: Table S2). CASE1 and her partner also had a normal distribution of T cell subpopulations (Additional file 1: Table S3), and both expressed comparable levels of CXCR4 and CCR5 on CD4^+^ lymphocytes (Additional file 1: Figure S4).

**Figure 3:**
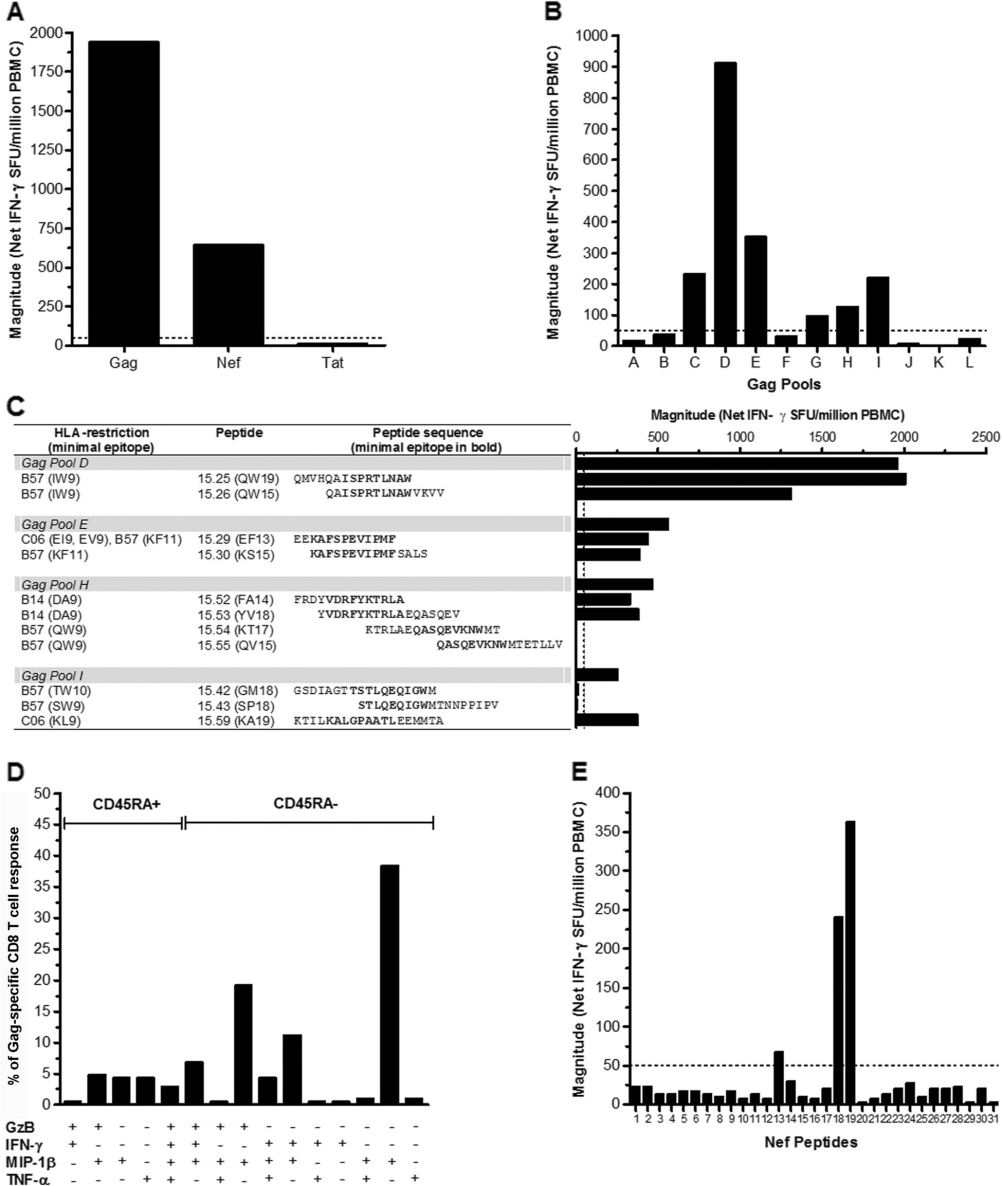
**T-cell responses of CASE1 T cells to Gag, Nef, and Tat peptides. (A)** Recognition of Gag and Nef, but not of Tat peptide pools by CASE1 PBMC collected in June 2011. **(B)** Recognition of Gag pools (A-L): pools D, E, G, H, and I contain well-defined HLA-B*57 restricted Gag epitopes. **(C)** Identification of epitopes recognized by CASE1 contained in Gag pools D, E, H and I. The table provides the HLA-restriction, the peptide designation and its sequence. The sequence of minimal epitopes for both Gag and Nef are shown in bold. **(D)** PBMC isolated from CASE1 were either left unstimulated or were stimulated with Gag peptide #25 and #26 and functionality of CD8+ T assessed by means of cytofluorimetric analysis for the production of Granzyme-B (GzB), Interferon-γ (IFN-γ), CC chemokine ligand 4 (CCL4)/Macrophage Inflammatory Protein-1β (MIP-1β) and Tumor Necrosis Factor-α (TNF-α); the cell population was subdivided into the CD45RA^+^ and CD45RA^neg^ CD8^+^ T-cell subsets and the percentages of these subsets were calculated relative to the peptide 25 and 26 Gag-specific response. **(E)** Recognition of Nef overlapping peptides. The HLA-B*57 and C*06 restricted minimal epitopes contained in Nef #13 (RPMTYKAAVDLSHFLK) were: RPMTYKAAV (C*06), MTYKAAVDL (C*06), KAAVDLSHF (B*57), AAVDLSHFL (C*06); minimal epitopes in Nef #18 (LDLWIYHTQGYFPDWQNY) and Nef #19 (YHTQGYFPDWQNYT) were: HTQGYFPDW (B*57) and GYFPDWQNY (C*06). The dotted line indicates the negative cut-off of the assay.

The breadth and magnitude of T-cell responses were assessed using CASE1 PBMC collected in Nov 2009 and June 2011. T cell responses were observed against Gag and Nef but not against Tat peptides (Figure 3A). Recognition of Gag peptide pools was mainly directed against regions containing well-defined HLA-B*57-restricted Gag epitopes (pools D, E, G, H and I, Figure 3B), without appreciable differences between the two dates.

Among the peptides present in Gag pools D and E, those expressing HLA-B*57 restricted epitopes (I/LW9 peptides 25 and 26 from pool D; KF11 peptide 29 and 30 pool E) mounted the strongest T-cell response (Figure 3C) as reported for LTNP [18]. Interestingly, recognition of the B*14-restricted epitope DA9, previously associated with virological control [19], was also observed (peptides 52 and 53 from pool H), whereas recognition of the B57* restricted epitope QW9 (peptide 54 and 55) was absent (Figure 3C). Moreover, recognition of pool I was not directed against the B*57-restricted epitope TW10 (peptides 42 and 43), but toward the C*06 restricted epitope KL9 (peptide 59, Figure 3C).

Among CD8^+^ T cells specific for HLA-B*57 (I/L)W9 restricted Gag epitopes (peptides 25 and 26), 34.6% expressed CCL4/MIP-1β and 50.5% were polyfunctional (Figure 3D). Recognition of Nef peptide pools was also mainly directed against regions containing well-defined HLA-B*57 and C*06 restricted Nef epitopes (Figure 3E).

## Conclusions

We here report that a woman (CASE1), who has been infected with HIV-1 for at least 20 years, has remained in good health with features of both EC and LTNP for the last 14 years. CASE1 co-expresses HLA-B (*14 and *57) alleles and HLA-B-mediated immune responses, together with HLA-C alleles (*06 and *08.02) associated with SNPs rs9264942C/C and rs67384697G/deletion and HLA-C restricted immune responses as her most evident features compatible with strong and durable control of HIV-1 infection and disease progression.

Although CASE1’s CD4^+^ T cells were infectable *in vitro* by both R5 and X4 HIV-1 strains, they showed lower levels of virus replication in comparison to those of the seronegative partner, suggesting the presence of restriction factors, such as p21 [20], limiting the virus replicative capacity in vitro and perhaps accounting for the lack of virus replication observed both *in vivo* and upon *ex vivo* cultivation of CASE1’s PBMC and gut-associated leukocytes.

Cell-associated HIV-1 DNA was detectable in PBMC and gut-mucosa derived cells at very low levels while *gag* sequences from single copy plasma RNA molecules and PBMC-associated HIV-1 DNA revealed the late emergence of mutations in two immunodominant HLA-B*57-restricted Gag epitopes co-existing on the same sequences. The mutation T242N observed in the TW10 epitope typically emerges early in infection and is known to effect CTL escape and impair viral fitness. However, this mutation is has not been associated with the EC status, being more frequently detected in B*57^+^ HIV-1 progressors [21]. The second mutation emerged in the I/LW9 B*57 associated epitope, restoring the original IW9 epitope with little or no impact on viral escape or fitness reported in prior studies. This behavior is reminiscent of EC who have extremely low levels of viral RNA in plasma, significant differences between contemporary viruses in the plasma and PBMC, and a high frequency of synonymous mutations in the plasma virus [22-25]. It is also noteworthy that HLA-B*57 epitope variation emerged in plasma-associated virus at the same sampling times of acquisition of two potential N-linked glycosylation sites in Env, including one that was previously implicated in the evasion of Ab neutralization [26]. These viral features, together with the persistence of CD8-mediated T cell responses, suggest the persistence of extremely low levels of virus replication in CASE1. The acquisition of several important amino acid substitutions in plasma-associated virus and in PBMC, in 2009 and 2011, respectively, suggests that her virological control may be waning.

In addition, CASE1 carries a unique combination of HLA-B protective alleles associated with CD8^+^ T cell-mediated control of HIV-1 replication and strong CD8^+^ T cells polyfunctional responses against HLA-B*57 and B*14-restricted Gag epitopes, as also observed in ELC and LTNP [27] that may account for the lack of CD4^+^ T cell depletion in peripheral blood and gut [28]. The higher levels of expression of HLA-C alleles carrying a deleted miR-148a/b binding site vs. those without a deleted site might facilitate greater T-cell recognition of infected cells [29]. In support of this interpretation CASE1 was also characterized by the presence of long lasting C*06-restricted T-cell responses against Nef epitopes.

HLA molecules play a central role in the control of HIV disease progression [17]. In this regard, CASE1 has multiple HLA features that were previously individually associated with control of HIV-1 viremia, including HLA class I alleles (HLA-B*057 and *014, HLA-C*06 and *0802) and two SNPs within the HLA-C locus (rs9264942C/C, and 263 del/del, 259 T/T, 261C/C, 266 T/T on rs67384697). Thus, co-expression of protective HLA-B and HLA-C alleles as well as of HLA-C SNPs, and the associated T cell immune responses, some of which polyfunctional [30], likely provide the strong and durable control of HIV-1 replication and disease progression in CASE1.

## Additional file

Additional file 1: Table S1. Titer of anti-HIV neutralizing Ab in CASE1’ serum and mucosal-derived fluid. **Table S2.** Percentage of B lymphocyte phenotypes in CASE1, her partner, other Elite controllers and HIV-1 negative controls. **Table S3.** Peripheral T lymphocyte phenotype distribution (%) in CASE1 and her partner. **Figure S1.** CASE1’s HCV related clinical features. **Figure S2.** CASE1’s intestinal mucosa histological and ultrastructural analysis. **Figure S3.** Amino acid alignments of CASE1 Gag (A) and Env-gp120 (B) proteins. **Figure S4.** CXCR4 and CCR5 expression by CASE1’s and partner’s peripheral blood CD4^+^ T cells.
