# Supplementary material for: Spontaneous control of HIV-1 viremia in a subject with protective HLA-B plus HLA-C alleles and HLA-C associated single nucleotide polymorphisms
Source: J Transl Med. 2014 Dec 5;12:335. doi: 10.1186/s12967-014-0335-6 (PMC4272524; doi:10.1186/s12967-014-0335-6)
Supplement: Additional file 1: Table S1. — Titer of anti-HIV neutralizing Ab in CASE1’ serum and mucosal-derived fluid. Table S2. Percentage of B lymphocyte phenotypes in CASE1, her partner, other Elite controllers and HIV-1 negative controls. Table S3. Peripheral T lymphocyte phenotype distribution (%) in CASE1 and her partner. Figure S1. CASE1’s HCV related clinical features. Figure S2. CASE1’s intestinal mucosa histological and ultrastructural analysis. Figure S3. Amino acid alignments of CASE1 Gag (A) and Env-gp120 (B) proteins. Figure S4. CXCR4 and CCR5 expression by CASE1’s and partner’s peripheral blood CD4+ T cells. [file 12967_2014_335_MOESM1_ESM.docx]

**Spontaneous Control of HIV-1 Viremia in a Subject with Protective HLA-B plus HLA-C Alleles and HLA-C associated Single Nucleotide Polymorphisms**

**Table of contents:**

1. Materials and Methods
2. Additional Tables
3. Additional Figures
4. Additional Figure Legends
5. References

**List of investigators:** Marco Moroni, Silvia Ghezzi, Paolo Baroli, Silvia Heltai, Davide De Battista, Simone Pensieroso, Mariangela Cavarelli, Stefania Dispinseri, Irene Vanni, Claudia Pastori, Pietro Zerbi, Antonella Tosoni, Elisa Vicenzi, Manuela Nebuloni, Kim Wong, Hong Zhao, Sarah McHugh, Guido Poli, Lucia Lopalco, Gabriella Scarlatti, Roberto Biassoni, James I. Mullins, Mauro S. Malnati, and Massimo Alfano.

1. **MATERIALS AND METHODS**

**Antibodies (Ab).** CD45RA FITC (clone ALB11) and CD8 ECD (clone SFCI21Thy2D3) were obtained from Beckman Coulter. CCR5 PE (clone 2D7/CCR5), CCL4/MIP-1β PE (clone D21-1351), TNF-α PE-Cy7 (clone MAb11), IFN-γ-Alexa700 (clone B27), FoxP3 (clone 259/C7), CD127 (clone hIL-7R-M21), CD27 (clone MT271), CD25 (clone 2A3), CCR7 (clone 3D12), CD3 APC-H7 (clone SK7), CD4 Pacific Blue (clone RPA-T4) and CD4 APC (clone RPA-T4) were obtained from Becton Dickinson. CXCR4 mAB (clone 12G5) was obtained from McKesson Clinical Services. LIVE/DEAD fixable Aqua dead cell stain kit and Granzyme B (GzB, clone GB11) were obtained from Invitrogen.

**Viral genetic analysis.** Plasma RNA was isolated using the QIAGEN QIAamp Viral RNA Mini Kit and PBMC DNA was isolated using the QIAGEN QIAamp DNA Mini Kit. Viral cDNA was synthesized using BlueScript (Takara) according the manufacturer’s instructions using RT2[[1](#_ENREF_1)], nef3[[2](#_ENREF_2)] and oligo dT as primers. Viral sequences in cDNA and PBMC DNA were PCR amplified following end-point dilution of template DNA to sequence individual viral templates without detection of PCR-associated errors and avoiding template resampling[[3](#_ENREF_3)]. Multiplex first-round PCR was performed with the Gag1 and RT2 primers[[1](#_ENREF_1)] to amplify an ~2,550bp fragment encompassing *gag* and the ED3[[4](#_ENREF_4)] and Nef3[[2](#_ENREF_2)] primers to amplify an ~3,080bp fragment encompassing *env*. Individual second round PCRs were performed with Gag2[[1](#_ENREF_1)] and RSP15R[[5](#_ENREF_5)] primers for an ~1,610bp *gag* amplicon and gp120-5 and gp120-3 primers[[6](#_ENREF_6)] for an ~1,605bp *env*-gp120 amplicon (including primers).

PCR products were excised from agarose gels and Sanger sequencing was performed at the High Throughput Genomics Center, Seattle, WA. Sequences were aligned using the MUSCLE[[7](#_ENREF_7)] option within SEAVIEW[[8](#_ENREF_8)] and phylogenetic trees were generated using the PhyML algorithm, GTR model of evolution and NNI tree sampling within DIVEIN[[9](#_ENREF_9)]. Output trees were visualized in FigTree1.4 (<http://tree.bio.ed.ac.uk/software/figtree/>) (Additional Figure 3). Sequences were deposited in Genbank and are available under the Accession numbers XX-YY.

**MiR-148a/b binding site (single nucleotide polymorphisms, SNP: rs67384697) and -35Kb 5’UTR HLA-C (SNP: rs9264942) analysis.** Genomic DNA was extracted from CASE1’s PBMC using the PureLink Genomic DNA kit (Invitrogen, Carlsbad CA). A pyrosequencing approach was used to determine SNPs: rs67384697 at residue-263 (NM_002117.4: c.^*^263delG) together with informative bases present at residues 256 (NM_002117.4: c.^*^256A>C), 259 (NM_002117.4: c.^*^259C>T), 261 (NM_002117.4: c.^*^261T>C) 266 (NM_002117.4: c.^*^266C>T), 267 (NM_002117.4: c.^*^267A>G) downstream from the HLA-C stop codon (3’UTR). Six hundred nM of the TgT CCA CCg TgA CCC CTg TC (miR148-frw2) and 100 nM of the 5’ biotinylated Agg gAA gTA AgA AgT TgC AgC TCA 3’ (5’biot- miR148-rev) oligodeoxynucleotides were used to generate a 138bp amplicon containing the miR-148a/miR-148b binding site. This fragment was sequenced using the miR148-sequencing primer 5’ TCT CCA TCT CTg TCT CAA 3’ at 800 nM. A similar approach was performed to study the rs9264942 (C/T) SNP localized 35kb 5’ upstream HLA-C gene using 200nM of both 5’ biotinylated-CCT gAg AAT TTT TgT CCC ACA Ag 3’ (5’biot-35kb/HLA-C frw) and 5’ TAA gTC AgA TTT TCC CgC TCC T 3’ (35kb/HLA-C rev) primers to amplify a 116bp fragment containing rs9264942. The SNP sequence was obtained in anti-sense orientation using the sequencing primer 5’ TCA gAA AgT CCC ACA gT 3’ at 800 nM. All amplifications were performed in a final volume of 25 μl using 25 ng of genomic DNA following a pre-amplification step of 2 min at 94 °C then 40 cycles of 15 sec at 94 °C, 30 sec at 60 °C and 30 sec at 72 °C with a final step of 1 min at 72 °C. Twenty-five μl of the PCR product was used for pyrosequencing. Sequencing by synthesis adds sequentially one base at a time from the sequencing primer following the sequence of the DNA complementary strand. The analysis of the miR148 binding site required the order of nucleotide dispensation containing the instrument selected control basis gAC gTC gAT CAg TgC TAg to detect the sequence [A/C]TT[C/T]A[T/C]g[g/deletion]Tg[C/T][A/g]C from the sequencing primer, while the analysis of the upstream sequence "35kb/HLA-C" required the dispensation order TgCTAgCAg to analyze the sequence gCCT[A/g]ggAAgTggg. Sequence analysis was performed and SNPs identified using the allele quantification (AQ) mode suite[[10](#_ENREF_10)].

**CCR5 and CCR2 genotyping.** CCR5/CCR5∆32 and CCR2/CCR2-64I genotyping were conducted on DNA extracted from pellets of 10^6^ PBMC. Cells were lysed with proteinase K, and their genomic DNA extracted with phenol/chloroform. The CCR5 sequence was amplified with the outer primer set as previously described[[11](#_ENREF_11)]. Cycling conditions were the following for both sets of primers: denaturation at 94°C for 10 min, followed by 24 cycles of 15 sec at 95°C, 30 sec at 55°C, and 30 sec at 72 °C each, and a final extension at 65°C for 5 min. The region overlapping the 32 bp sequence deleted in the CCR5∆32 allele was amplified by PCR as described[[12](#_ENREF_12)]. PCR amplification products were analyzed by agarose gel electrophoresis to discriminate by size the full-length product from CCR5∆32.

Since the CCR2-64I and the wild-type alleles do not differ in length, nor can they be discriminated by restriction fragment length polymorphism, they were analyzed with a TaqMan-based allelic discrimination assay as previously described[[13](#_ENREF_13)].

**Histological and ultrastructural analysis of intestinal biopsies.** Three intestinal biopsies (from rectum, sigmoid and descending colon) were fixed in 10% neutral formalin and paraffin-embedded. The histological diagnoses were performed following routine histochemical staining [hematoxylin–eosin, periodic acid Schiff (PAS) and Giemsa] of 3 µm sections. Three biopsies from same sites were also fixed in 2.5% buffered glutaraldehyde, post-fixed in 1% osmium tetroxide, and embedded in epoxy resin (Durcupan^TM^ ACM Fluka, Germany). Ultrathin sections were stained with uranyl acetate and lead citrate and examined by transmission electron microscopy (Additional Figure 2).

**ELISpot assay for IFN-γ.** Ninety-six-well plates (MAIPS4510, Millipore, Bedford, MA, USA) were coated overnight at 4°C with IFN-γ-specific capture Ab (clone 2G1, Thermo Scientific Pierce, Rockford, IL, USA) diluted in sterile Dulbecco’s phosphate buffered saline (D-PBS, Lonza-BioWhittaker, Verviers, Belgium) at 2 µg/ml. After blocking for 1h with complete RPMI medium [10% FBS (Lonza-BioWhittaker) in RPMI (Lonza-BioWhittaker)], the plates were washed 3 times with PBS. Single/pools of HIV-1 derived peptides were plated in each well. After 4h, PBMC (≥ 80% viable) in complete RPMI medium were added to each well at a concentration of 200,000 PBMC/well. PBMC were plated in the presence or absence of a mixture of co-stimulatory Ab composed of anti-CD28 and anti-CD49d at a concentration of 1.3 µg/ml each (BD Biosciences, San Jose, CA, USA). The cells were then incubated at 37 °C for 18h in a 5% CO_2_ incubator. After stimulation, the detection Ab (mouse anti-human IFN-γ-biotin clone B133.5, Thermo Scientific Pierce, Rockford, IL, USA) was diluted in PBS and added at a final concentration of 2 µg/ml for 1h at room temperature. Subsequently, wells were washed 6 times with wash buffer (Thermo Scientific Pierce) and 3 times with PBS. Alkaline phosphatase conjugated streptavidin (Amersham Pharmacia Biotech Europe GmbH, Freiburg, Germany) was added to each well followed by incubation for 1 h at room temperature. Chromogenic substrate (NTB [Nitrotetrazolium Blue Chloride] and BCIP [5-Bromo-4-chloro-3-indolyl phosphate p-toluidine salt], Sigma, St. Louis, MO) was added to each well for 5 min and blocked by 3 washes with water. A mixture made by Phorbol 12-Myristate 13-Acetate (PMA) and Ionomycin (I) (Sigma), at the concentration of 1 ng/ml and 500 ng/ml, respectively, was used as positive control to obtain at least 500 SFU per million cells. PBMC suspended in complete RPMI medium alone and seeded in duplicate were used as a negative control. Responses were considered positive when the number of SFU per million cells in stimulated wells, subtracted of the values of negative control wells, was higher than thresholds (≥30 for Tat antigens and was ≥50 for Gag and Nef antigens). The SFU were counted with a computer-assisted ELISpot image analyzer (Eli-Scan, A.EL.VIS, Hannover, Germany), using the software Eli.Analyse V4.2 (A.EL.VIS).

**Intracellular cytokine staining.** Thawed PBMC (≥ 80% viable) were plated in a 96-well plate after 4 h of resting at a concentration of 1x10^6^ PBMC/well in complete RPMI medium [10% FBS (Lonza-BioWhittaker) in RPMI (Lonza-BioWhittaker)] with single or pools of HIV-1 derived peptides (2 µM) in the presence of a mixture of co-stimulatory Ab composed of anti-CD28 and anti-CD49d at a concentration of 1.3 μg/ml each (Becton Dickinson). The cells were then incubated at 37°C for 5 h in a 5% CO_2_ incubator. The GolgiPlug protein transport inhibitor (Becton Dickinson) was added to each well after 1h of incubation at a concentration of 10 µg/ml. For each individual donor a sample without peptides was included to calculate background staining. Stimulated cells were then suspended in PBS (Lonza-BioWhittaker) and incubated with LIVE/DEAD fixable Aqua dead cell stain (Invitrogen) to assess their viability. After washing with FACS Buffer [1% FBS (Lonza-BioWhittaker) 0.01% Na Azide (Sigma) in PBS (Lonza-BioWhittaker)], cells were fixed and permeabilized using BD Cytofix/Cytoperm Kit (Becton Dickinson). Then the fluorochrome-conjugated Abs were added to the cell suspension and incubated for 30 min at 4°C. After washing, samples were acquired with a Gallios flow cytometer (Beckman Coulter, Inc.) and analyzed using FlowJo version 8.8.7 (Tree Star, Ashland, Oregon). Lymphocytes were gated on a forward scatter area vs. side scatter area using a pseudo-color dot plot and dead cells were removed according to Aqua stain. CD8^+^CD4^-^ T-cells were identified within the CD3^+^ lymphocytes. Then, the functional markers (CCL4/MIP-1β, IFN-γ, TNF-α and GzB) were plotted versus forward scatter and all positive cells were gated. A mock, unstimulated sample, was used to identify the background in each subject for each cytokine. After background subtraction, the 90^th^ percentile of the negative values, both in percentage and event counts, was calculated and these two values were considered as thresholds. Samples were considered positive when producing percentage or event counts higher than both thresholds. Boolean gating analysis identified 16 patterns by calculating every possible combination of the 4 populations gated. Response patterns with at least 1 positive to CCL4/MIP-1β or IFN-γ or TNF-α stain were taken into consideration for analysis, thus resulting in a total of 28 immune response patterns. The frequency of the total CD8^+^ T cell response was calculated by summing the frequency of each response pattern and the percentage of the total CD8^+^ T cell response was calculated by dividing the percentage of each population by the frequency of the total responses.

**Purification and quantification of immunoglobulins (Ig).** Affinity purification of total Ig, IgA and IgG fractions from serum and genital fluids were carried out through a sequential automatic chromatography system (Biologic Duoflow, BIO-RAD Laboratories, Hercules, CA, USA), as previously described[[14](#_ENREF_14)]. Briefly, IgG fractions were purified by affinity chromatography on HiTrap Protein G HP columns (GE-Healthcare). Fractions were then eluted with 8 volumes of 200 mM Glycine buffer (pH 2.0; 0.2 mL/min flow) and neutralized at pH 7.0 with 1 M Tris-HCl buffer pH 11.0. The unbound fraction containing IgA and IgM was recovered, processed and purified by anion exchange column chromatography. After a dialysis step in binding buffer A (40 mM NaCl, 20 mM Tris-HCl pH 7.2) fractions were applied onto HiTrap Q HP column (GE-Healthcare). IgA and IgM fractions were eluted from the column with 10 volumes of buffer containing 340 mM NaCl, 20 mM Tris-HCl, pH 7.2. IgA-IgM Ab were concentrated on Amicon cartridges and underwent a further gel filtration step on a Bio-Silect SEC 400-5 column (BioRad), to separate IgA from IgM Ab. Samples were eluted in PBS buffer (flow: 1 mL/min) and fractions collected and sterilized by passage through 0.22 µ membranes before storage at -80°C.

Purified mucosal IgA and IgG were measured by ELISA. Briefly, ELISA plates were coated with a 1:2,000 dilution of a goat anti-human IgA or IgG (100 µl/well) in coating buffer and incubated for 1 h at 37°C. After washing, blocking buffer (1% Skim Milk in Phosphate Buffer, Sigma) was added and plates were incubated for 1 h at 37 °C. Serial dilutions of samples and IgA or IgG reference standards (Sigma) were incubated for 1 h at 37 °C. After washing, Goat anti-human IgA-Biotin (diluted 1:5,000) or Goat anti-human IgG-Biotin conjugate diluted 1:2,000 (KPL) was added and incubated for 1 h at room temperature. Streptavidin-HRP conjugate (Vector Laboratory) diluted 1:3,000 was then added and incubated for 1 h at room temperature. TMB substrate (KPL) was incubated for 5 min at room temperature in the dark. Subsequently, H_2_SO_4_ (10%) was added and plates were read with a spectrophotometer at 450 nm. Total IgA or IgG concentrations were determined by interpolation, using the calibration line of IgA or IgG reference standards, respectively.

**TZM-bl neutralization assay.** Neutralizing activity of heat inactivated serum, cervico vaginal washes (CV), seminal plasma (SP) and purified Ig was evaluated using a panel of 6 pseudoviruses (obtained through the NIBSC, Centre for AIDS Reagents, UK) including four Clade B (QH0692, AC10, 6535 and PVO), one Clade C (ZM214) and one Clade B laboratory strain (SF162), in a standardized and validated single-round infection assay. Stocks of HIV-1 Env pseudoviruses were produced by co-transfecting 293T/17 cells with 2 µg of an HIV-1 *rev/env* expression plasmid and 12 µg of an *env*-deficient HIV-1 backbone plasmid (pSG3DEnv) using Lipofectamine transfection reagent (Invitrogen). Pseudovirus-containing supernatant was harvested 24 h following transfection, clarified by centrifugation and filtered through 0.45 mm filters, and single-use 1 ml aliquots were stored at -80 °C. The 50% tissue culture infectious dose (TCID_50_) for each pseudovirus preparation was determined by infection of TZM.bl cells.

The CCR5- and CD4-transfected TZM.bl cell line (JC53-bl obtained through the NIH AIDS Research and Reference Reagent Program, USA) was used as the target for HIV-1 neutralization assay, as previously described. Briefly, 3-fold serial dilutions of each sample were plated in duplicate (96-well flat bottom plate) in 10% D-MEM growth medium (100 ml/well). 200 TCID_50_ of each pseudovirus were added to each well in a volume of 50 ml and incubated for 1 h at 37 °C. TZM.bl cells were then added (1x10^4^/well in a 100 ml volume) in 10% D-MEM growth medium containing DEAE-dextran (Sigma) at a final concentration of 11 µg/ml. Assay controls included replicate wells of TZM.bl cells alone (cell control) and TZM.bl cells with virus (virus control). Following 48-h incubation at 37°C, 150 µl of culture medium were removed from each well and replaced with 100 µl of Bright-Glo luciferase reagent (Promega). After 2 min incubation, 150 µl of the cell lysate was transferred to a 96-well black solid plate and luminescence was measured using a Victor Light 2030 luminometer (Perkin Elmer). The 50% inhibitory dose (IC_50_) of heat inactivated serum, cervico vaginal washes, seminal plasma and purified Ig was calculated as the sample dilution that induced a 50% reduction in relative luminescence units vs. infected but untreated cells. A pool of 2F5, b12 and 2G12 neutralizing mAb (trimAb) was used at 7.4, 2.5, 0.82 and 0.27 µg/ml as positive control. A pool of five non HIV-related human sera, a pool of cervico vaginal washes from three healthy women and a pool of seminal plasma from 3 healthy men were used as negative controls. Results are shown as IC_50_ value per each sample (Additional Table 1).

**B cell phenotype.** Multi-color flow cytometry was performed on PBMC, acquiring 10^6^ formaldehyde-fixed cells with a FACS Gallios (Beckman Coulter, CA, USA) flow cytometer. The following mouse anti-human fluorochrome-conjugated mAb (BD, NJ, USA) were used: CD19 PERCP-Cy5.5 (clone SJ25C1), CD27 V450 (clone M-T271), CD21 PE (clone B-ly4) and CD10 PE-Cy7 (clone HI101). Dead cells were excluded using the Live/Death Vivid detection kit labeled with a near-infrared dye (Invitrogen, Carlsbad, CA, USA). Only singlets were acquired and live B-cells were gated on Vivid-CD19^+^ lymphocytes; transitional B-cells were identified as CD19^+^CD27^+^CD10^+^. Thereafter, the CD10^neg^ cell population was gated and naive (CD27^neg^CD21^+^), resting memory (CD27^+^CD21^+^), zctivated memory (CD27^+^CD21^neg^) and tissue-like memory (CD27^neg^CD21^neg^) B cells were identified. Analyses were performed using FlowJo software version 8.8.3 (Three Star Inc.).

**Anti-HIV neutralizing Ab responses in PBMC assay.** The detailed protocol for virus titration and neutralization of a PBMC-based assay is available in the EUROPRISE website ([www.europrise.org](http://www.europrise.org)). Briefly, the TCID_50_ was determined by incubating six 5-fold viral supernatant dilutions, starting from 1/5, with 10^5^ PHA-activate PBMC from 2 healthy donors in 5 replicates. Cells were washed after 1 and 3 days of culture and fresh medium was added. At day 7, supernatant from each well was tested for the presence of HIV-1 p24 Gag antigen by an in house ELISA (Aalto Bio Reagents, Dublin). TCID_50_ was determined with the Reed-Munch calculation.

Sera from 2 follow-up time points were tested for neutralizing activity against a panel of 6 heterologous viruses: SF162 (subtype B, R5), 654 (subtype B, R5), 634 (subtype B, R5), J213 (subtype B, R5X4), DU174 (subtype C, R5), and CM244 (subtype CRF01, R5). Viruses 654 and 634 were primary isolates from an EC and a chronically infected adult, respectively, whereas J213 was from a child in chronic infection. Viral supernatants were incubated with 4-fold serum dilutions (from 1/20 to 1/1280) in a 96-well plate for 1h at 37°C. Thereafter, 10^5^ PHA-stimulated PBMC from 2 healthy donors were added to each well and further processed as described for the titration assay. At day 7 each well was tested for the presence of HIV-1 p24 Gag antigen by ELISA. Neutralization was evaluated at 90% inhibition of the input virus.

1. **SUPPLEMETARY TABLES**

**Supplementary Table 1. Titer of anti-HIV neutralizing Ab in CASE1’ serum and mucosal-derived fluid.**

| **Virus** | **Clade** | **Tropism** | **TCID_50_** | **Target cells** | **Specimen** | **CASE1** | **TrimAb** | **Negative controls** |
| --- | --- | --- | --- | --- | --- | --- | --- | --- |
| **SF162** | B | R5 | 200 | TZM.bl | Serum | 960 | <0.25 | <20 |
|  |  |  | 12 | PBMC | Serum | 36 | 2.7 | <20 |
|  |  |  | 200 | TZM.bl | Purified serum IgG | >190 | <0.25 | >190 |
|  |  |  | 200 | TZM.bl | Purified serum IgA | >26 | <0.25 | >50 |
|  |  |  | 200 | TZM.bl | Purified mucosal IgG | >71 | <0.25 | CVL>71; SP >150 |
|  |  |  | 200 | TZM.bl | Purified mucosal IgA | >25 | <0.25 | CVL>25; SP>76 |
| **CM244** | CRF01 | R5 | 43 | PBMC | Serum | <20 | 42.6 | <20 |
| **QH0692** | B | R5 | 200 | TZM.bl | Serum | <20 | 2 | <20 |
|  |  |  | 200 | TZM.bl | Purified serum IgG | >190 | 2 | >190 |
|  |  |  | 200 | TZM.bl | Purified serum IgA | >26 | 2 | >50 |
| **6535** | B | R5 | 200 | TZM.bl | Serum | <20 | 0.08 | <20 |
| **PV10** | B | R5 | 200 | TZM.bl | Serum | <20 | <0.25 | <20 |
| **AC10** | B | R5 | 200 | TZM.bl | Serum | <20 | 0.46 | <20 |
| **654** | B | R5 | 87 | PBMC | Serum | <20 | >50 | <20 |
| **634** | B | R5 | 41 | PBMC | Serum | <20 | 3.62 | <20 |
| **ZM214** | C | R5 | 200 | TZM.bl | Serum | <20 | 9.09 | <20 |
| **DU174** | C | R5 | 28 | PBMC | Serum | <20 | >25 | <20 |
| **J213** | B | R5X4 | 32 | PBMC | Serum | <20 | 42.6 | <20 |

Values in the last 3 columns are expressed as IC_50_, corresponding to sample dilution/concentration equal to 50% reduction of infection. Values of sera are expressed as dilution 1/n, whereas purified Ab and trimAb as µg/ml. Negative controls (healthy, HIV-1 negative individuals) are represented by pool of 5 sera, seminal plasma and 3 cervico vaginal lavage (CVL).

**Supplementary Table 2. Percentage of B lymphocyte phenotypes in CASE1, her partner, other Elite controllers and HIV-1 negative controls.**

| **B cell populations** | **CASE1** | **Partner** | **Elite Controllers**  **Median value (min-max)** | **Controls**  **Median value (min-max)** |
| --- | --- | --- | --- | --- |
| Total | 6.72 | 11.1 | n.a. | 9.98 (2.85-22.4) |
| Naïve | 78.6 | 62.1 | 52.52 (21.9-78.6) | 67.61 (34-92) |
| Resting-Memory | 16.6 | 36.2 | 24.97 (8.77-49.8) | 21.69 (5.11-49) |
| Activated-Memory | 2.45 | 0.88 | 11.64 (2.45-29.1) | 4.40 (0.43-19.4) |
| Tissue-like memory | 2.35 | 0.81 | 10.87 (2.35-22.8) | 6.21 (0.81-21.7) |
| Transitional | 5.87 | 3.39 | 5.42 (0-15.4) | 3.62 (0.31-9.43) |

B lymphocytes were gated as reported[[15](#_ENREF_15)] and B cell subpopulations identified based on the expression of the following antigens: Total; CD19^+^, Naïve; CD19^+^CD27^neg^CD21^+^, Resting-Memory; CD19^+^CD27^+^CD21^+^, Activated-Memory; CD19^+^CD27^+^CD21^Low^, Tissue-like memory; CD19^+^CD27^neg^CD21^Low^, and Transitional; CD19^+^CD10^+^. Percentages of B cell phenotypes of CASE1 and her partner are compared to median values from 18 HIV-1 infected Elite Controllers and 95 HIV-uninfected (control) individuals, as reported[[15](#_ENREF_15)]. n.a; not available.

**Supplementary Table 3. Peripheral T lymphocyte phenotype distribution (%) in CASE1 and her partner.**

| **T cell populations** | **CASE1** | **Partner** |
| --- | --- | --- |
| Naïve (CD45RA^+^CCR7^+^CD27^+^) | 38 | 37.9 |
| Central Memory (CD45RA^neg^CD27^+^CCR7^+^) | 48 | 51.9 |
| Transitional Memory (CD45RA^neg^CD27^+^CCR7^neg^) | 10 | 4.8 |
| Effector Memory (CD45RA^neg^CCR7^neg^CD27^neg^) | 2 | 1 |
| Terminally Differentiated (CD45RA^+^CD27^neg^CCR7^neg^) | 1 | 0 |
| Treg (FoxP3^+^CD25^+^) | 6 | 6.15 |
| Th17 (CCR6^+^IL-17^+^) | 0.6 | 1.03 |

T lymphocytes were gated based on the expression of the cell surface markers indicated between brackets. Values are in agreement with those reported for other cohorts of EC[[16](#_ENREF_16)].

1. **SUPPLEMENTARY FIGURES**

**Supplementary Figure 1**

**
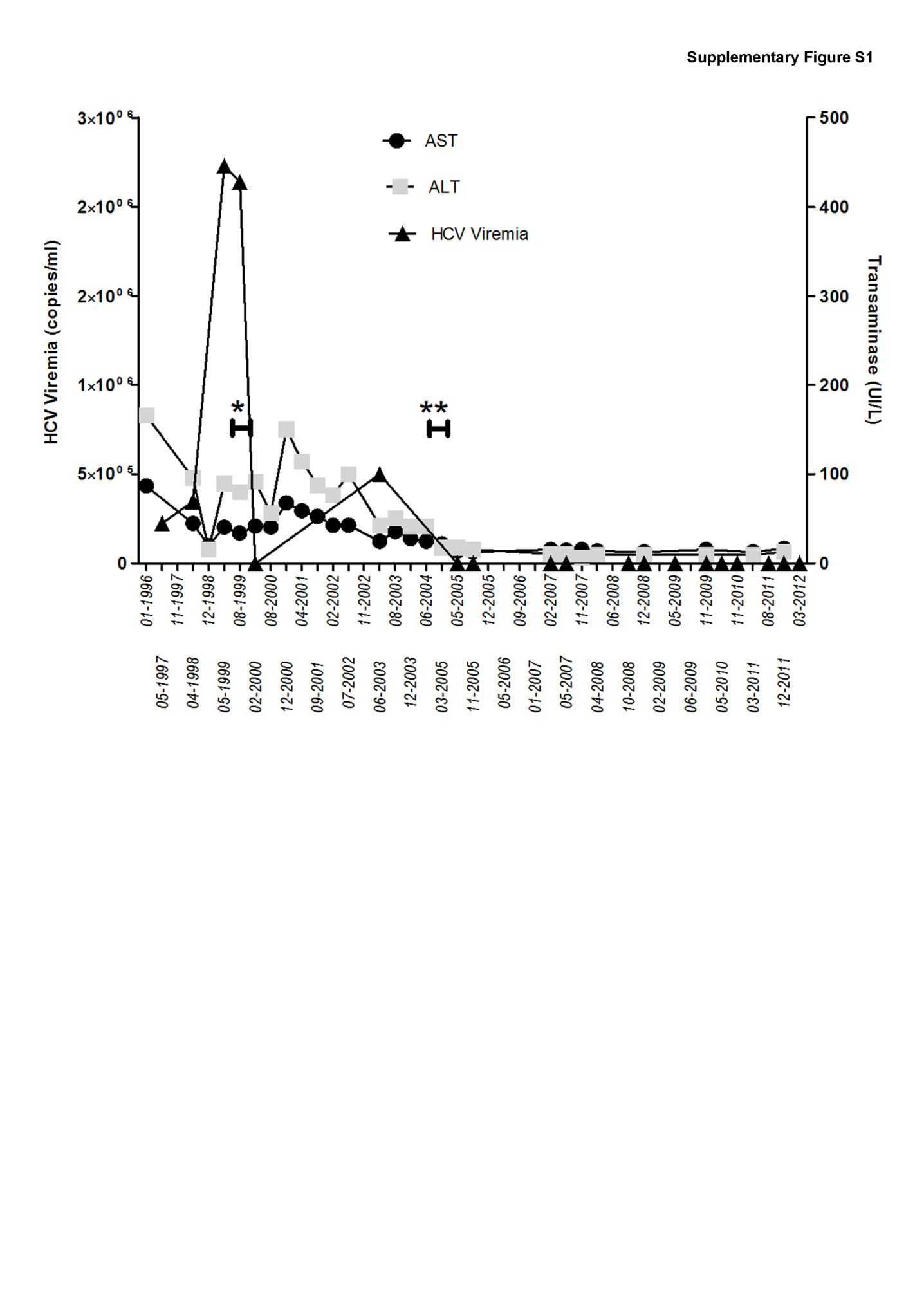
**

**
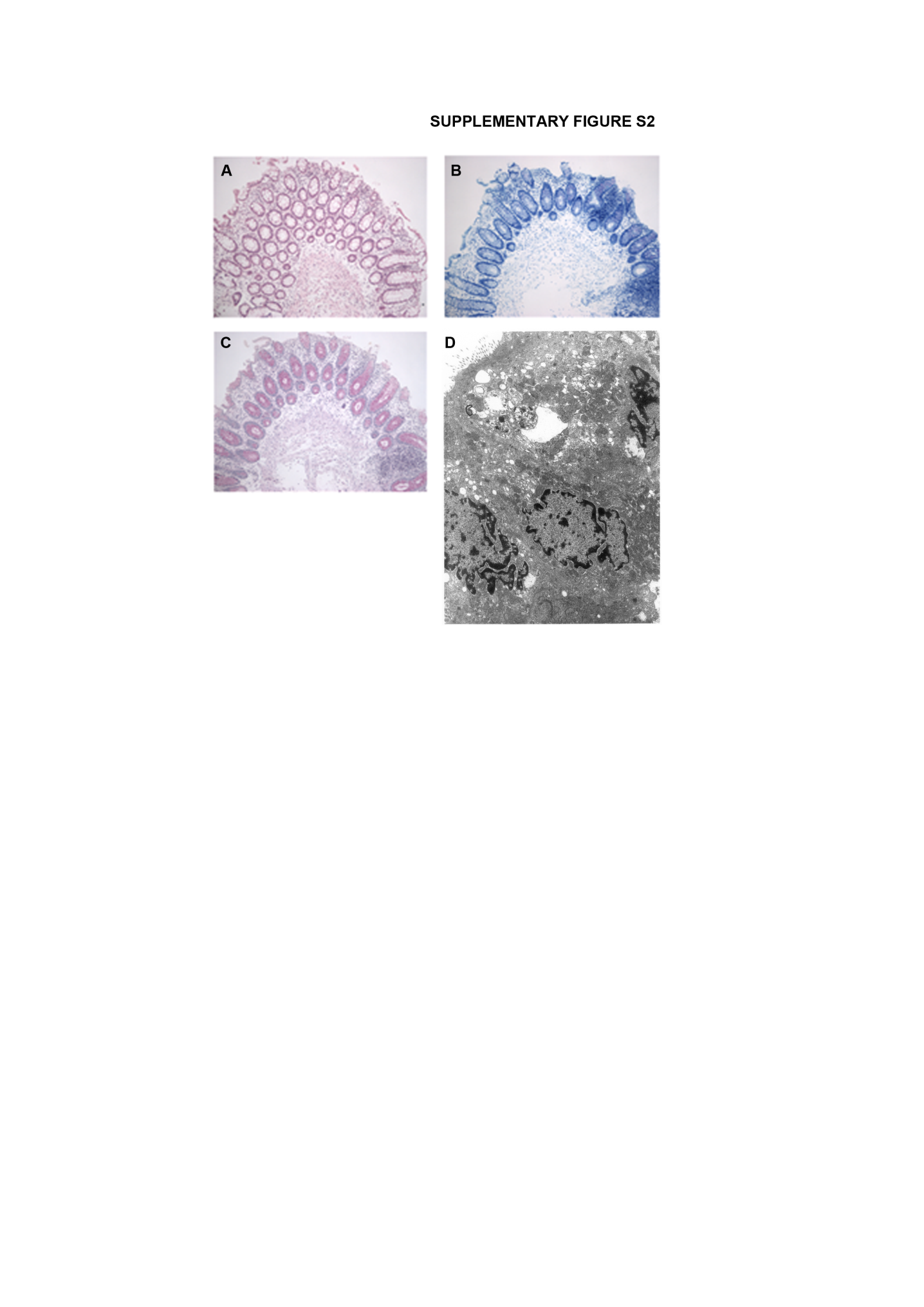
 Supplementary Figure 2**

**
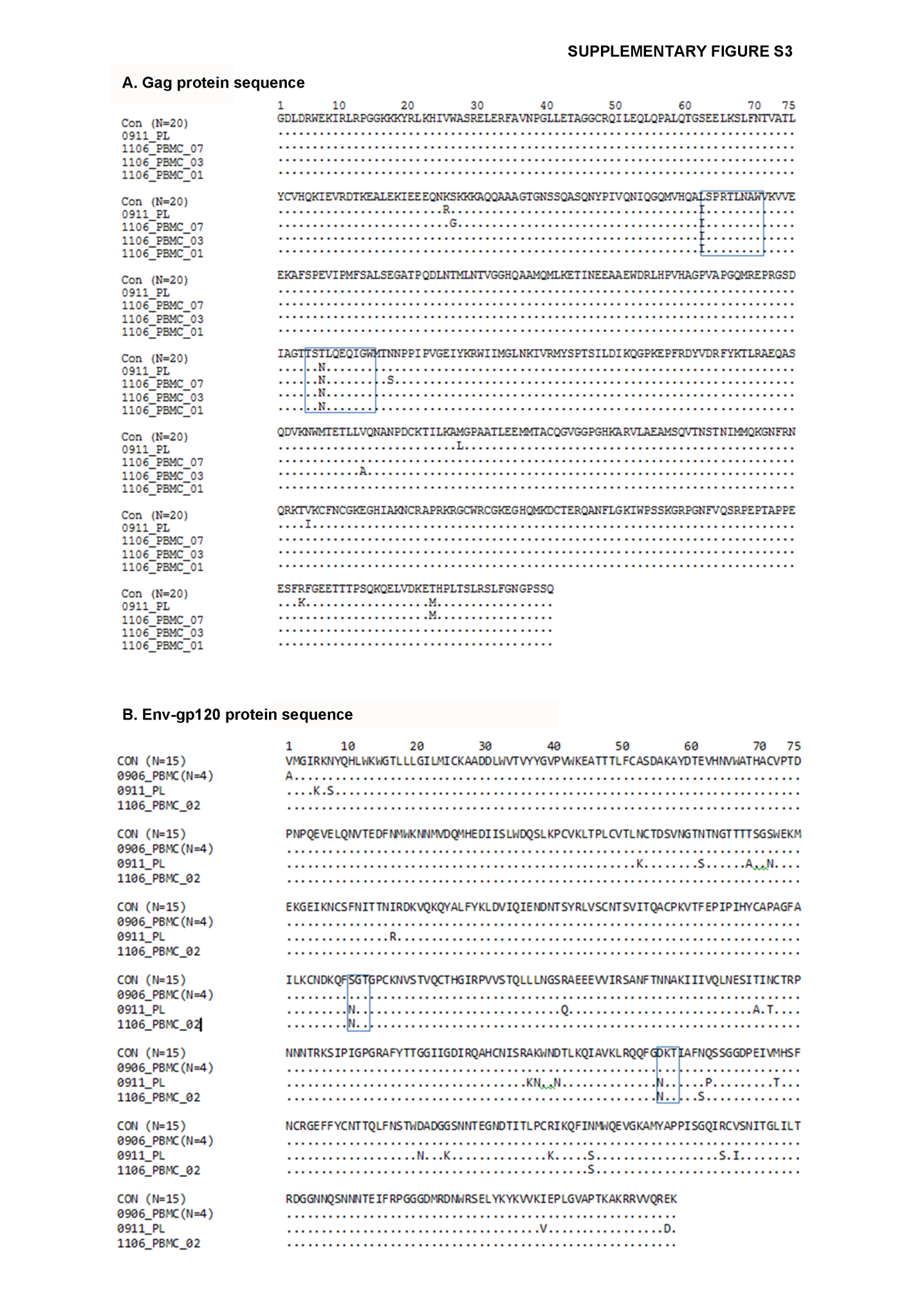
 Supplementary Figure 3**

**Supplementary Figure
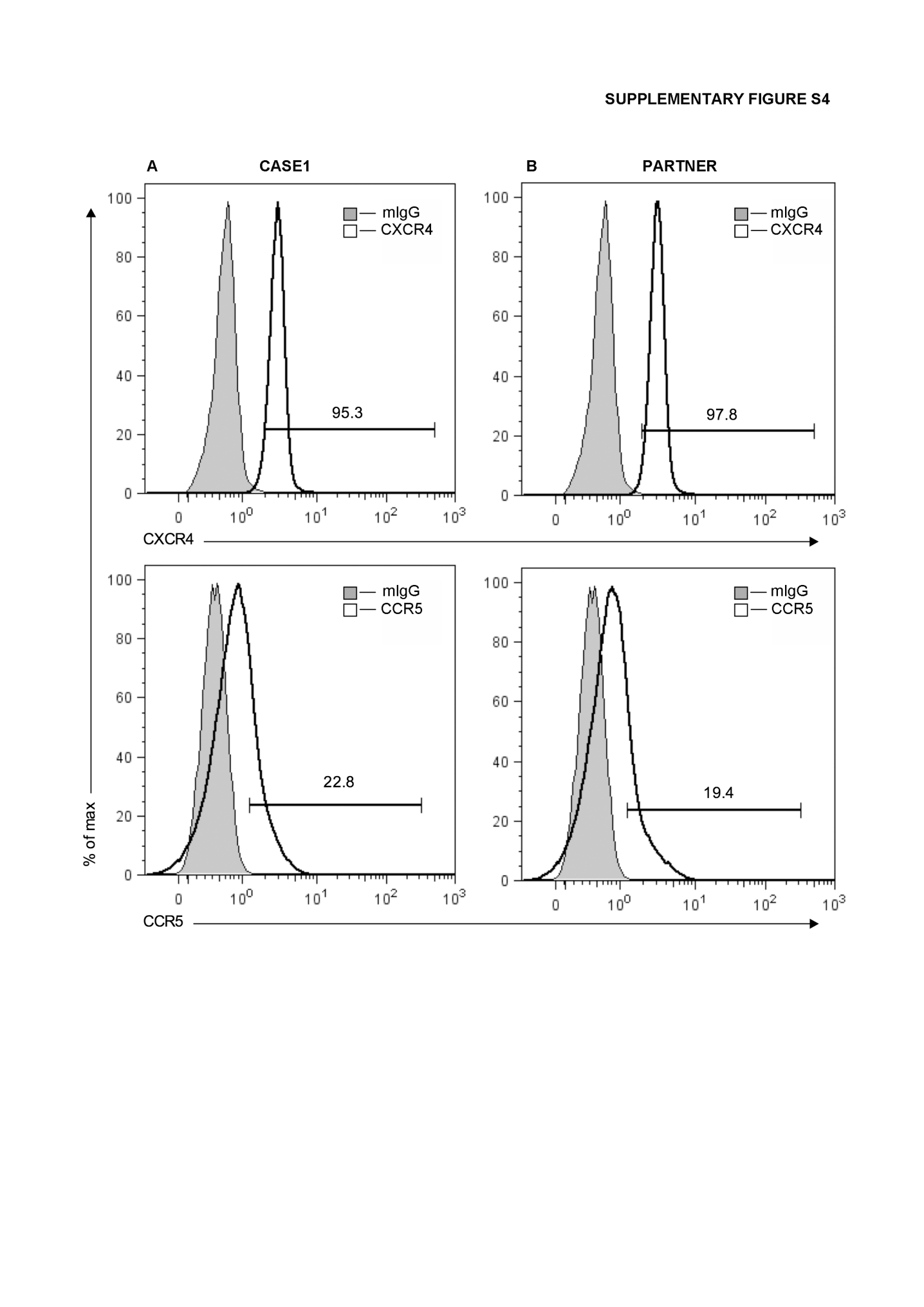
44. SUPPLEMENTARY FIGURE LEGENDS**

**Supplementary Figure 1. CASE1’s HCV related clinical features.** In 1998, CASE1 was diagnosed with HCV infection (genotype 3a), with a viral load ranging from 2 to 5x10^5^ copies/ml and with a liver biopsy showing aggressive chronic hepatitis with moderate phlogosis and fibrosis. Treatment with Interferon-α (3 times a week for 6 months) was not successful, as high HCV viremia persisted at the end of treatment (≈ 2x10^6^ copies/ml). A liver biopsy performed in 2004 revealed light fibrogenic activity (grading score by Knodell-Desmet[[17](#_ENREF_17)]: 5) and portal fibrosis (staging score by Knodell-Desmet: 1). An anti-HCV therapy with Pegylated Interferon (80 mg weekly) plus Rebetol (600 mg a day for 6 months) was then introduced. CASE1 has been negative for HCV RNA since 2005 with normal levels of transaminases. Asterisks indicate periods of anti-HCV therapies, either with Interferon-α (*) or with Pegylated Interferon plus Rebetol (**). AST; aspartate aminotransferase, ALT; alanine aminotransferase, HCV; Hepatitis C Virus.

**Supplementary Figure 2. CASE1’s intestinal mucosa histological and ultrastructural analysis.** Biopsies of colon were evaluated for histological morphology, identification of inflammatory infiltrates and microbes (A-C, magnification: 10x) and ultrastructural morphology (D, magnification: 3,000x). Transmission electron microscopy did not reveal the presence of virions nor of cell injury (as evident by the lack of tubule-reticular intra-cytoplasmic inclusions), and only mild signs of nonspecific enterocyte alterations (i.e., increased number and enlarged volume of lysosomal bodies and microvilli with irregular shape) were noted.

**Supplementary Figure 3. Amino acid alignments of CASE1 Gag (A) and Env-gp120 (B) proteins.** Dots (.) indicate homology to the consensus (CON) sequences from this patient’s viruses. The Gag alignment begins at position 11 of the Gag protein sequence, whereas the Env alignment covers the entire gp120 protein. N=x in the sequence name indicates the number of sequences encoding the identical protein sequences – only one sequence from each group is shown. Sequences without (N=x) in their names were only detected once each. Boxes are drawn around features in Gag corresponding to the B57*IW9 and TW10 epitopes and two PNLGS in Env [Corresponding to the NX(T/S) motif].

**Supplementary Figure 4. CXCR4 and CCR5 expression by CASE1’s and partner’s peripheral blood CD4^+^ T cells.** Lymphocytes were purified from Ficoll-derived PBMC and the expression of HIV-1 co-receptors on CD4^+^ T cells was measured by cytofluorimetric analysis; overlaid histograms show the surface expression of CXCR4 (left) and CCR5 (right) by CD4^+^ T lymphocytes (grey histograms show the isotype control while white histograms show the percentage of positive cells).

**5. SUPPLEMENTARY MATERIAL REFERENCES**

1. Liu Y, McNevin J, Cao J, Zhao H, Genowati I, Wong K, McLaughlin S, McSweyn MD, Diem K, Stevens CE, et al: **Selection on the human immunodeficiency virus type 1 proteome following primary infection.** *J Virol* 2006, **80:**9519-9529.

2. Frenkel LM, Mullins JI, Learn GH, Manns-Arcuino L, Herring BL, Kalish ML, Steketee RW, Thea DM, Nichols JE, Liu SL, et al: **Genetic evaluation of suspected cases of transient HIV-1 infection of infants.** *Science* 1998, **280:**1073-1077.

3. Rodrigo AG, Goracke PC, Rowhanian K, Mullins JI: **Quantitation of target molecules from polymerase chain reaction-based limiting dilution assays.** *AIDS Res Hum Retroviruses* 1997, **13:**737-742.

4. Delwart EL, Shpaer EG, Louwagie J, McCutchan FE, Grez M, Rubsamen-Waigmann H, Mullins JI: **Genetic relationships determined by a DNA heteroduplex mobility assay: analysis of HIV-1 env genes.** *Science* 1993, **262:**1257-1261.

5. Liu Y, Holte S, Rao U, McClure J, Konopa P, Swain JV, Lanxon-Cookson E, Kim M, Chen L, Mullins JI: **A sensitive real-time PCR based assay to estimate the impact of amino acid substitutions on the competitive replication fitness of human immunodeficiency virus type 1 in cell culture.** *J Virol Methods* 2013, **189:**157-166.

6. Liu Y, Curlin ME, Diem K, Zhao H, Ghosh AK, Zhu H, Woodward AS, Maenza J, Stevens CE, Stekler J, et al: **Env length and N-linked glycosylation following transmission of human immunodeficiency virus Type 1 subtype B viruses.** *Virology* 2008, **374:**229-233.

7. Edgar RC: **MUSCLE: a multiple sequence alignment method with reduced time and space complexity.** *BMC Bioinformatics* 2004, **5:**113.

8. Galtier N, Gouy M, Gautier C: **SEAVIEW and PHYLO_WIN: two graphic tools for sequence alignment and molecular phylogeny.** *Comput Appl Biosci* 1996, **12:**543-548.

9. Deng W, Maust BS, Nickle DC, Learn GH, Liu Y, Heath L, Kosakovsky Pond SL, Mullins JI: **DIVEIN: a web server to analyze phylogenies, sequence divergence, diversity, and informative sites.** *Biotechniques* 2010, **48:**405-408.

10. Guindon S, Gascuel O: **A simple, fast, and accurate algorithm to estimate large phylogenies by maximum likelihood.** *Syst Biol* 2003, **52:**696-704.

11. Romiti ML, Colognesi C, Cancrini C, Mas A, Berrino M, Salvatori F, Orlandi P, Jansson M, Palomba E, Plebani A, et al: **Prognostic value of a CCR5 defective allele in pediatric HIV-1 infection.** *Mol Med* 2000, **6:**28-36.

12. Morawetz RA, Rizzardi GP, Glauser D, Rutschmann O, Hirschel B, Perrin L, Opravil M, Flepp M, von Overbeck J, Glauser MP, et al: **Genetic polymorphism of CCR5 gene and HIV disease: the heterozygous (CCR5/delta ccr5) genotype is neither essential nor sufficient for protection against disease progression. Swiss HIV Cohort.** *Eur J Immunol* 1997, **27:**3223-3227.

13. Kostrikis LG, Huang Y, Moore JP, Wolinsky SM, Zhang L, Guo Y, Deutsch L, Phair J, Neumann AU, Ho DD: **A chemokine receptor CCR2 allele delays HIV-1 disease progression and is associated with a CCR5 promoter mutation.** *Nat Med* 1998, **4:**350-353.

14. Donadoni C, Bisighini C, Scotti L, Diomede L, Ngyen M, Nouhin J, DeSantis L, Zambon A, Ferrari D, Gallotta G, et al: **Setting of methods for analysis of mucosal antibodies in seminal and vaginal fluids of HIV seropositive subjects from Cambodian and Italian cohorts.** *PLoS One* 2010, **5:**e9920.

15. Pensieroso S, Galli L, Nozza S, Ruffin N, Castagna A, Tambussi G, Hejdeman B, Misciagna D, Riva A, Malnati M, et al: **B-cell subset alterations and correlated factors in HIV-1 infection.** *AIDS* 2013.

16. Chase AJ, Yang HC, Zhang H, Blankson JN, Siliciano RF: **Preservation of FoxP3+ regulatory T cells in the peripheral blood of human immunodeficiency virus type 1-infected elite suppressors correlates with low CD4+ T-cell activation.** *J Virol* 2008, **82:**8307-8315.

17. Desmet VJ, Gerber M, Hoofnagle JH, Manns M, Scheuer PJ: **Classification of chronic hepatitis: diagnosis, grading and staging.** *Hepatology* 1994, **19:**1513-1520.
